# ICOS is required for the generation of both central and effector CD4^+^ memory T‐cell populations following acute bacterial infection

**DOI:** 10.1002/eji.201445421

**Published:** 2015-04-07

**Authors:** Clare L. Marriott, Gianluca Carlesso, Ronald Herbst, David R. Withers

**Affiliations:** ^1^ Institute for Biomedical ResearchCollege of Medical and Dental SciencesUniversity of BirminghamUK; ^2^ RespiratoryInflammation and AutoimmunityResearch DepartmentMedImmune LLCGaithersburgMDUSA

**Keywords:** ICOS, CD28, CD4^+^ Memory, T‐cell responses, Tfh

## Abstract

Interactions between ICOS and ICOS ligand (ICOSL) are essential for the development of T follicular helper (Tfh) cells and thus the formation and maintenance of GC reactions. Given the conflicting reports on the requirement of other CD4^+^ T‐cell populations for ICOS signals, we have employed a range of in vivo approaches to dissect requirements for ICOS signals in mice during an endogenous CD4^+^ T‐cell response and contrasted this with CD28 signals. Genetic absence of ICOSL only modestly reduced the total number of antigen‐specific CD4^+^ T cells at the peak of the primary response, but resulted in a severely diminished number of both T central memory and T effector memory cells. Treatment with blocking anti‐ICOS mAb during the primary response recapitulated these effects and caused a more substantial reduction than blocking CD28 signals with CTLA4Ig. During the memory phase of the response further signals through ICOS or CD28 were not required for survival. However, upon secondary challenge only Tfh cell expansion remained heavily ICOS‐dependent, while CD28 signals were required for optimal expansion of all subsets. These data demonstrate the importance of ICOS signals specifically for memory CD4^+^ T‐cell formation, while highlighting the potential of therapeutically targeting this pathway.

## Introduction

Memory is a fundamental aspect of the adaptive immune response that enables rapid responses against pathogens upon reencounter. Memory T cells are the product of successful T‐cell responses, existing within long‐lived populations that last many months in mice 1 and years in humans 2. T cells of any given specificity are rare 3, requiring extensive proliferation and differentiation to form a pool of effector CD4^+^ T cells. The proliferation and differentiation of naive CD4^+^ T cells requires both TCR/MHCII interactions and costimulatory signals initially through CD28 4, 5, although subsequently many other receptor/ligand pairs can contribute 6. This results in effector T‐cell subsets with distinct functions and developmental requirements 7. Evidence that memory cells develop from effector cells, rather than as a separate population derives from observations that cytokine producing and nonproducing effector cells generate memory cells equally well 1, 8, 9. The memory cell pool can be divided into two subsets based upon chemokine receptor expression and cytokine secretion; T central memory (Tcm) cells express CCR7, required for trafficking through secondary lymphoid tissue and need to proliferate to produce cytokines other than IL‐2. T effector memory (Tem) cells lack CCR7 expression and thus are thought to be unable to recirculate through secondary lymphoid tissue; rather these cells can rapidly produce effector cytokines such as IFN‐γ as well as IL‐2 upon antigen recognition 10, 11.

The signals that control the development and subsequent survival of the memory cell pool require further understanding. The cytokines IL‐7 and IL‐15 are known to be important for the homeostasis of CD4^+^ memory T cells, while TCR/MHCII interactions are not thought to be required 12. Whether cell surface molecules provide additional survival signals is unclear. Such experiments are technically challenging since signals required for the formation of the memory cell population must be distinguished from signals required for persistence. Impaired CD4^+^ memory T‐cell survival in mice where group 3 innate lymphoid cells (ILC3s) were absent suggests that costimulatory molecules expressed by these cells such as OX40 ligand may support memory cells 13, however, this has not been determined in vivo. Previous work indicated that OX40 expression was tightly linked with antigen exposure 14 rather than consistently expressed by memory CD4^+^ T cells, suggesting other surface molecules may play a role in their survival once formed.

CD28 family members regulate T‐cell responses through costimulatory and inhibitory signals 15, 16. Previously, it has been shown that CD28 and ICOS are essential for productive primary CD4^+^ T‐cell responses, however they each have distinct roles 17, 18. CD28 is constitutively expressed on CD4^+^ T cells and is required to enhance T‐cell proliferation and IL‐2 production 19, 20. CD80^−/−^CD86^−/−^ mice, which lack both ligands of CD28, show impairments in isotype class switching and are unable to form GC 21. ICOS is upregulated on CD4^+^ T cells following antigenic stimulation 17, 18, 22, 23 and engagement with ICOS ligand (ICOSL) provides a key signal required for the generation of T follicular helper cells (Tfh) and consequently the formation and maintenance of GC 24, 25. Thus deficiency in ICOS also results in a reduction in class switched antibody and B‐cell memory 22, 23, 26. Studies in CD28‐deficient mice demonstrated the importance of ICOS signals to CD4^+^, but not CD8^+^ T‐cell responses to viral and parasite infections 27. Recently the T cells of patients with ICOS deficiency were characterized in detail and found to be deficient in both Tcm and Tem cell populations, indicating that ICOS signals are fundamental to the generation and/or the survival of these cells 28. However, studies in mice have indicated ICOS to be important specifically for the generation or survival of Tem cells 29, 30, 31, although this relied heavily on identifying Tem cells based only on CD44 and CD62L expression and the use of unphysiological numbers of TCR transgenic T cells. A recent study tracking endogenous antigen‐specific CD4^+^ T cells revealed a requirement for ICOS signals only in the Tcm population 11.

Therefore we sought to dissect the requirements of a polyclonal CD4^+^ T‐cell population for CD28 and ICOS signals during the different stages of a T‐cell response. Using MHCII tetramers an endogenous CD4^+^ T‐cell population recognizing 2W1S peptide was tracked, since such cells will most closely reflect the size and polyclonality of the naive T cell pool in human patients. Our aim was to determine at what points in a CD4^+^ T‐cell response CD28 and ICOS signals were required, using a combination of blocking mAb and genetically deficient mice to enable WT memory cells formed in vivo to be assessed. Genetic deficiency in ICOSL or blockade of ICOS in the primary response resulted in a grossly impaired memory cell pool with both Tcm and Tem cells reduced. The subsequent survival of these cells did not require further signals through ICOS and only the Tfh cell population was dependent on ICOS signals upon secondary challenge. While memory CD4^+^ T cells did not require further signals through CD28 for survival, all subsets of T cells were at least partially dependent on CD28 signals for expansion upon secondary challenge. Collectively our data indicate an essential role for ICOS in the formation of all memory cells and highlight the potential of therapeutic targeting of this pathway to block CD4^+^ T‐cell responses.

## Results

### ICOS, as well as CD28, is required for optimal formation of antigen‐specific CD4^+^ T cells

**Figure 1:**
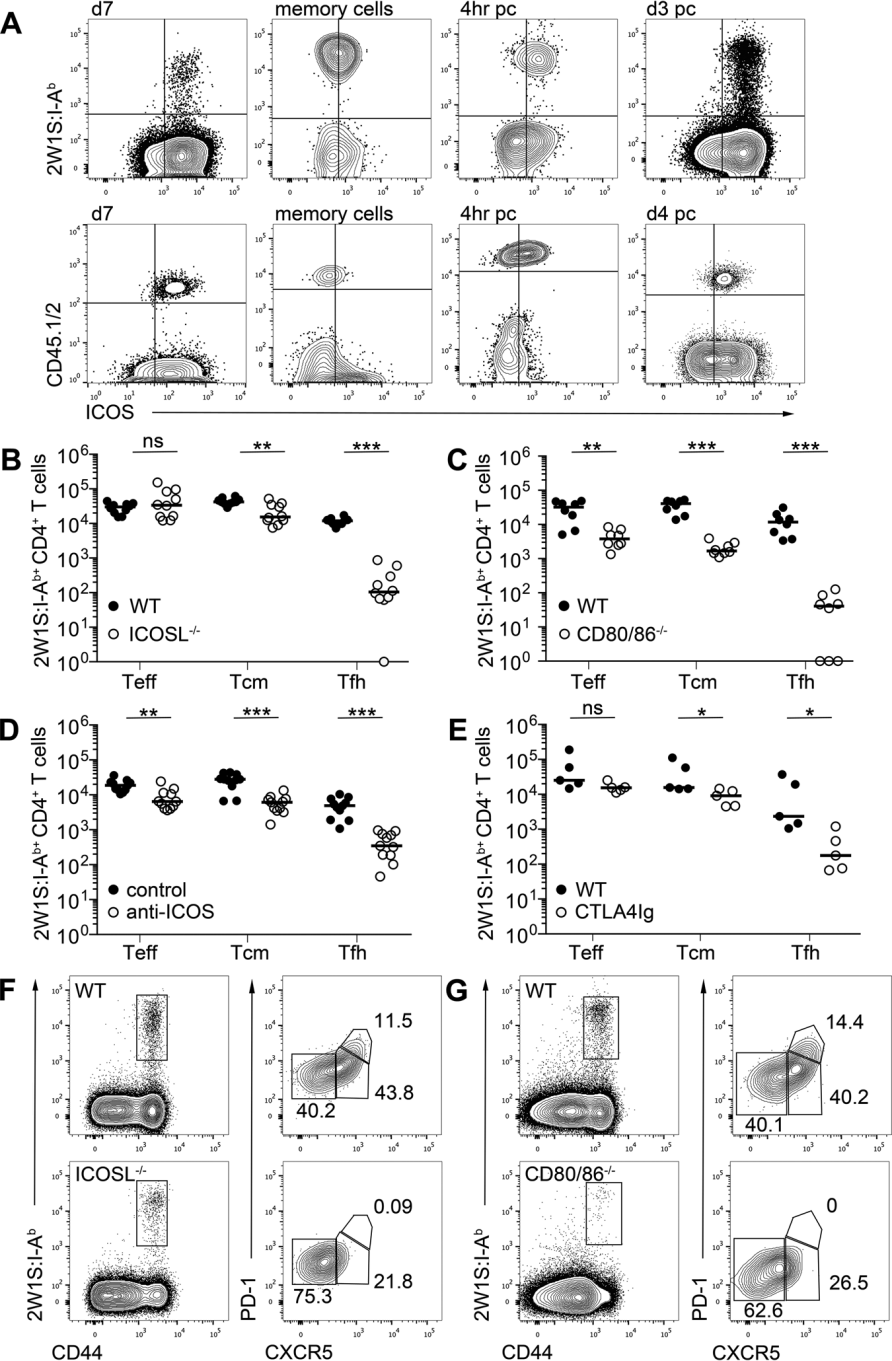
ICOS, as well as CD28, is required for optimal formation of antigen‐specific T cells in a primary response. WT mice were infected with Lm‐2W1S and 2W1S‐specific T cells were detected by flow cytometry. (A) (top) Expression of ICOS on 2W1S:I‐A^b+^ CD4^+^ T cells and (bottom) TCR transgenic SM1 T cells at 7 dpi, memory cells, 4 h and 3 (2W1S:I‐A^b+^) or 4 (SM1) days postsecondary challenge. Plots are gated on CD3^+^ B220^−^CD11b^−^CD11c^−^ followed by CD4^+^CD44^hi^ T cells. (B–E) Enumeration of CD44^hi^ 2W1S:I‐A^b+^ CD4^+^ CXCR5^−^ (Teff), CXCR5^+^ (Tcm precursors), and CXCR5^+^PD^−^1^+^ (Tfh) cells 7 dpi in (B) ICOSL^−/−^ mice; (C) CD80^−/−^CD86^−/−^ mice; (D) C57Bl/6 mice treated with anti‐ICOS or isotype control mAb 0 and 3 dpi; and (E) CTLA4Ig mice. (F and G) Expression pattern of CXCR5 and PD‐1 by CD44^hi^ 2W1S:I‐A^b+^ CD4^+^ T cells in (F) ICOSL^−/−^ mice and (G) CD80^−/−^CD86^−/−^ mice. (A, F, and G) Plots are representative of ≥6 mice from two independent experiments in all experiments except (A) ICOS expression on SM1 cells 7 dpi and 4 h post secondary challenge, which represent two mice. Values on plots are percentages. (B–E) Graphs show pooled data from two independent experiments, each data point represents one mouse. Bars show medians. Values of 0 were assigned as 1. Mann–Whitney test: ns *p* > 0.05, *p ≤ 0.05, **p ≤ 0.01, ***p ≤ 0.001.

To dissect the precise requirements for CD28 and ICOS signals during a CD4^+^ T‐cell response, we used the 2W1S model pioneered by the Jenkins laboratory to enable an endogenous, polyclonal population to be assessed. Mice were infected with an attenuated *Listeria monocytogenes* expressing 2W1S peptide (Lm‐2W1S) as previously defined 1, and the 2W1S‐specific CD4^+^ T‐cell population was tracked from primary proliferation through to resting memory cells using MHCII tetramers. In this model, expression of OX40 is closely linked to antigen exposure and tightly regulated 14. Naïve CD44^lo^ 2W1S‐specific CD4^+^ T cells lacked ICOS expression (Supporting Information Fig. 1), however, at all subsequent time points after activation (day 7, memory cells, 4 h postsecondary challenge and day 3 post secondary challenge) ICOS was expressed by all, or the majority of responding cells (Fig. 1A). Expression of ICOS was limited to CD44^hi^ CD4^+^ T cells and virtually absent in mice lacking the ligands of CD28 (Supporting Information Fig. 1). Expression of ICOS on responding TCR transgenic CD4^+^ T cells was also assessed, using low numbers of SM1 T cells to model the population size of endogenous naive T‐cell pools 32. ICOS expression was comparable with the endogenous CD4^+^ T‐cell population except on memory SM1 cells, which expressed lower levels of ICOS than the memory 2W1S‐specific population (Fig. 1A). Following Lm‐2W1S infection, three subsets of CD4^+^ T cells can be defined: CXCR5^−^PD‐1^−^T‐bet^+^ effector cells (Teff) that give rise to Tem cells, CXCR5^+^PD‐1^−^Bcl‐6^+^ central memory precursors that give rise to Tcm cells and CXCR5^+^PD‐1^+^Bcl‐6^+^ Tfh cells 11. The Tcm precursor cell subset of 2W1S‐specific CD4^+^ T cells was significantly reduced in ICOSL^−/−^ mice 7 dpi (days postinfection) with Lm‐2W1S and Tfh cells were almost absent (Fig. 1B and F), consistent with previous studies of this response 1, 11. CD80^−/−^CD86^−/−^ mice were also analyzed 7 dpi as a comparison and all T‐cell subsets were significantly decreased in the absence of signaling through CD28 (Fig. 1C and G). We also sought to assess the effect of reagents blocking these interactions to explore therapeutic targeting of these pathways within the context of this response. Blocking anti‐ICOS mAb were administered at 0 and 3 dpi before mice were analyzed 7 dpi with Lm‐2W1S. Notably, this mAb recapitulated the effects seen in the ICOSL^−/−^ mice, with Tfh cell formation most significantly affected; although reductions in both the Tcm precursors and Teff cell subsets were also observed (Fig. 1D). To assess blockade of CD28 signals we investigated the 2W1S‐specific response in mice that express a CTLA4Ig fusion protein, blocking CD28 binding to CD80 and CD86, analogous to abatacept therapy. Serum levels of 10–30 μg/mL CTLA4Ig are maintained in the CTLA4Ig mice 33. Interestingly, while Tfh cell formation was substantially impaired, consistent with an inability to form GC within these mice 33, Teff and Tcm precursor populations were only modestly reduced compared with the CD80^−/−^CD86^−/−^ mice, consistent with an incomplete block of available CD28 ligands (Fig. 1E). Supporting the partial blockade of CD28 signals in these mice, numbers of Treg cells are substantially reduced in CTLA4Ig mice, compared with WT controls, although not to the numbers detected in CD80^−/−^CD86^−/−^ mice (Supporting Information Fig. 2).

### ICOS is required for the formation of both Tcm and Tem cells

**Figure 2:**
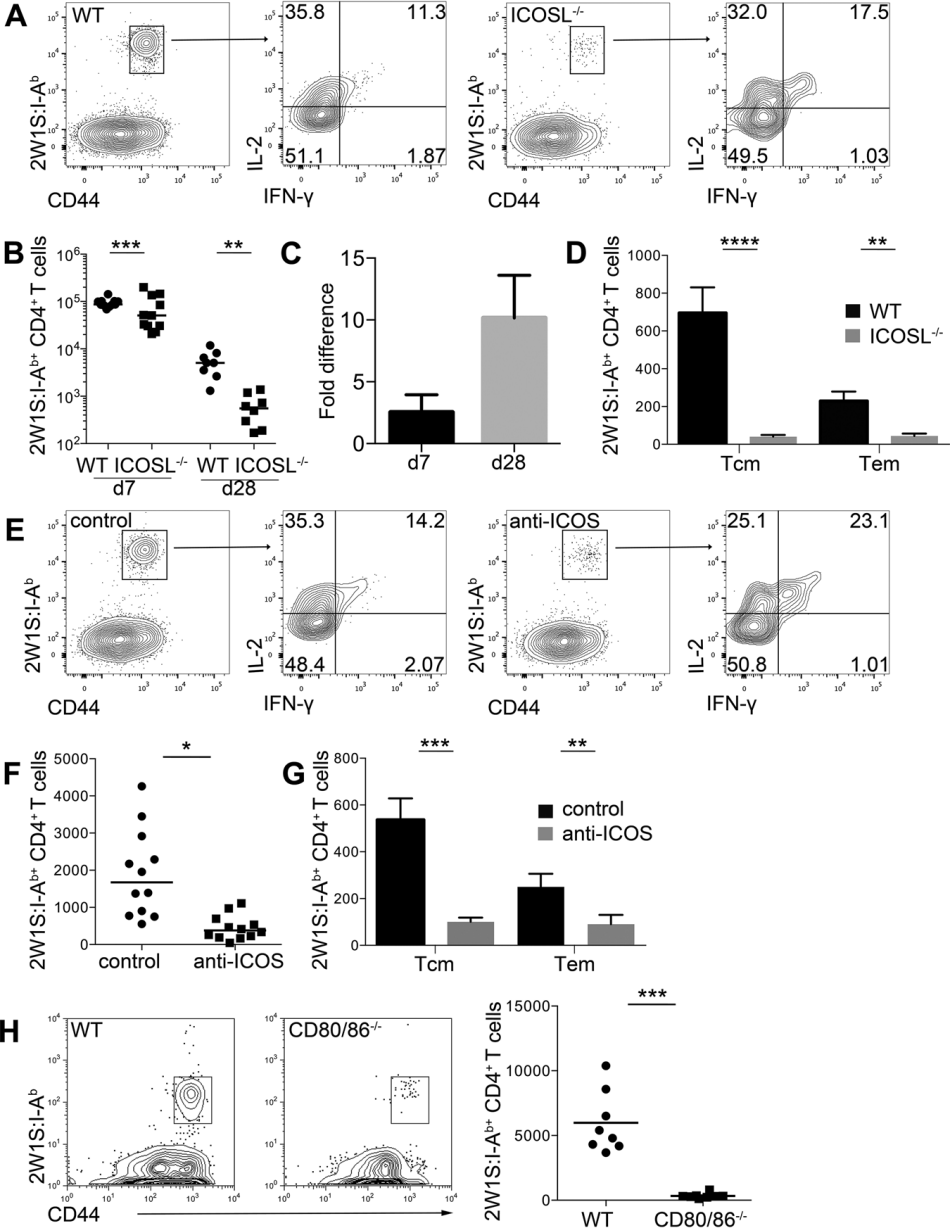
ICOS is required to form both Tcm and Tem cell pools. (A–D) WT and ICOSL^−/−^ mice were infected with Lm‐2W1S then restimulated in vivo 28 dpi with 2W1S peptide. (A) CD44^hi^ 2W1S:I‐A^b+^ memory CD4^+^ T cells were stained for cytokines IL‐2 and IFN‐γ and analyzed by flow cytometry. (B) Total number of CD44^hi^ 2W1S:I‐A^b+^ CD4^+^ T cells recovered at 7 and 28 dpi from WT and ICOSL^−/−^ mice; bars show median. (C) Fold difference between WT and ICOSL^−/−^ mice in number of CD44^hi^ 2W1S:I‐A^b+^ CD4^+^ T cells at 7 and 28 dpi. Bars show mean + SEM of fold difference between experiments. (D) CD44^hi^2W1S:I‐A^b+^ CD4^+^ memory T cells with Tcm (IL‐2^+^IFNγ^−^) or Tem (IL‐2^+^IFNγ^+^) phenotype were enumerated by flow cytometry from WT and ICOSL^−/−^ mice. Bars show mean + SEM from 12 mice. (E–G) WT mice were infected with Lm‐2W1S then restimulated in vivo 28 dpi with 2W1S peptide after administration of anti‐ICOS or isotype control mAb at 0 and 4 dpi. (E) CD44^hi^2W1S:I‐A^b+^ memory CD4^+^ T cells were stained for IL‐2 and IFN‐γ and analyzed by flow cytometry. (F) Total number of CD44^hi^ 2W1S:I‐A^b+^ CD4^+^ memory T cells recovered at 28 dpi; bars show medians. (G) Tcm and Tem phenotype cells were enumerated by flow cytometry. Bars show mean + SEM from 12 mice. (H) WT and CD80^−/−^CD86^−/−^ mice were infected with Lm‐2W1S then restimulated in vivo 28 dpi with 2W1S peptide. (Left) Representative flow cytometry plots and (right) total number of CD44^hi^ 2W1S:I‐A^b+^ CD4^+^ memory T cells recovered at 28 dpi. (A, E, and H) Plots are representative of ≥6 mice from ≥2 independent experiments, values on plots are percentages. (B–D and F–H) Graphs show pooled data from ≥2 independent experiments. Each data point represents one mouse. Mann–Whitney test: *p ≤ 0.05, **p ≤ 0.01, ***p ≤ 0.001, ****p ≤ 0.0001.

Formation of a memory CD4^+^ T‐cell pool is dependent upon a robust primary response 32, 34. Studies of ICOS^−/−^ and ICOSL^−/−^ mice have concluded a specific loss of Tem cells 29, 30, but following Lm‐2W1S infection of chimeras reconstituted with an equal mix of ICOS‐deficient and WT bone marrow cells, a reduced population of Tcm precursors was detected 11. Therefore we sought to assess the 2W1S‐specific memory cell pool in WT or ICOSL^−/−^ mice 28 dpi with Lm‐2W1S. To functionally identify Tcm and Tem cells, mice were restimulated in vivo with 2W1S peptide prior to analysis. Strikingly, the number of 2W1S‐specific memory cells was substantially reduced in ICOSL^−/−^ mice (Fig. 2A and B), with an approximate tenfold reduction in total number at this time point, compared with an approximate 3.5‐fold difference at day 7 (Fig. 2C). Notably, both the Tcm and Tem populations were heavily depleted compared with controls (Fig. 2D) in contrast to previous reports that observed effects only on the Tem cells 11, 29, 30. This impaired memory cell formation was also seen when anti‐ICOS mAb were administered to WT mice at 0 and 4 dpi (Fig. 2E–G). As expected, in CD80^−/−^ CD86^−/−^ mice, the memory cell pool was barely detected (Fig. 2H). These data indicate that signals through ICOS are essential in the formation of a robust memory cell compartment despite modest effects on total numbers of cells at the peak of the primary response.

### CD28 and ICOS signals are not required for antigen‐specific memory T‐cell survival

**Figure 3:**
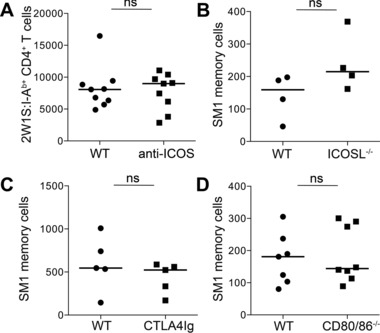
ICOS and CD28 costimulation are not required for antigen‐specific memory cell survival. (A) WT mice were infected with Lm‐2W1S, treated with blocking anti‐ICOS or isotype control mAb twice weekly for 4 weeks and 28 dpi CD44^hi^ 2W1S:I‐A^b+^ CD4^+^ memory T cells were enumerated by flow cytometry. (B–D) SM1 memory cells generated in WT mice were transferred into different hosts: (B) ICOSL^−/−^ mice; (C) CTLA4Ig mice; and (D) CD80^−/−^CD86^−/−^ mice and the number of surviving memory cells enumerated 4 weeks posttransfer by flow cytometry. (A and D) Graphs show pooled data from two independent experiments. (B, C) Graphs show representative data of two independent experiments. Each data point represents one mouse; bars show medians. Mann–Whitney test: ns *p* > 0.05.

Whether memory CD4^+^ T cells require signals through ICOS and CD28 for continued survival during the memory phase of the response has not been robustly tested. To circumvent problems associated with analyzing memory cell populations in mice where priming may be impaired, WT mice were infected with Lm‐2W1S and left for 4 weeks to form a 2W1S‐specific memory population. Blocking anti‐ICOS or isotype control mAb were given at 28 dpi twice weekly for 4 weeks, then the memory cell population was assessed. There was no significant difference in the number of 2W1S‐specificmemory cells recovered (Fig. 3A) indicating that signals through ICOS were not required to maintain the memory cell pool at this stage of the response. Since ICOS mAb blockade may be incomplete, we sought a second method by which WT memory cell survival could be assessed. Given the challenge of transferring tetramer specific CD4^+^ T‐cell populations, an allotype marked TCR transgenic SM1 memory population was generated in vivo by transferring 10^4^ naive SM1 cells into WT hosts that were then infected with attenuated *Listeria monocytogenes* expressing Flic (Lm‐Flic), thus keeping the agent with which the mice were infected consistent. Through transferring low numbers of TCR transgenic T cells, a physiological CD4^+^ T‐cell response is recapitulated 32. After 4 weeks, the SM1 cells were enriched and transferred into new hosts. Analysis of the memory SM1 cells confirmed their expression of ICOS at this time (Fig. 1A). Approximately 2.5 × 10^4^ SM1 memory cells were transferred and mice were analyzed 4 weeks posttransfer. Survival of WT SM1 memory cells was not impaired in ICOSL^−/−^, CD80^−/−^CD86^−/−^, or CTLA4Ig mice indicating that signals through B7 costimulatory molecules are not required for the continued survival of antigen‐specific memory cells once formed (Fig. 3B‐D). Therefore while formation of a robust memory population is highly dependent upon both CD28 and ICOS, the persistence of these cells does not require these signals.

### ICOS is required for expansion of antigen‐specific Tfh cells post secondary challenge

**Figure 4:**
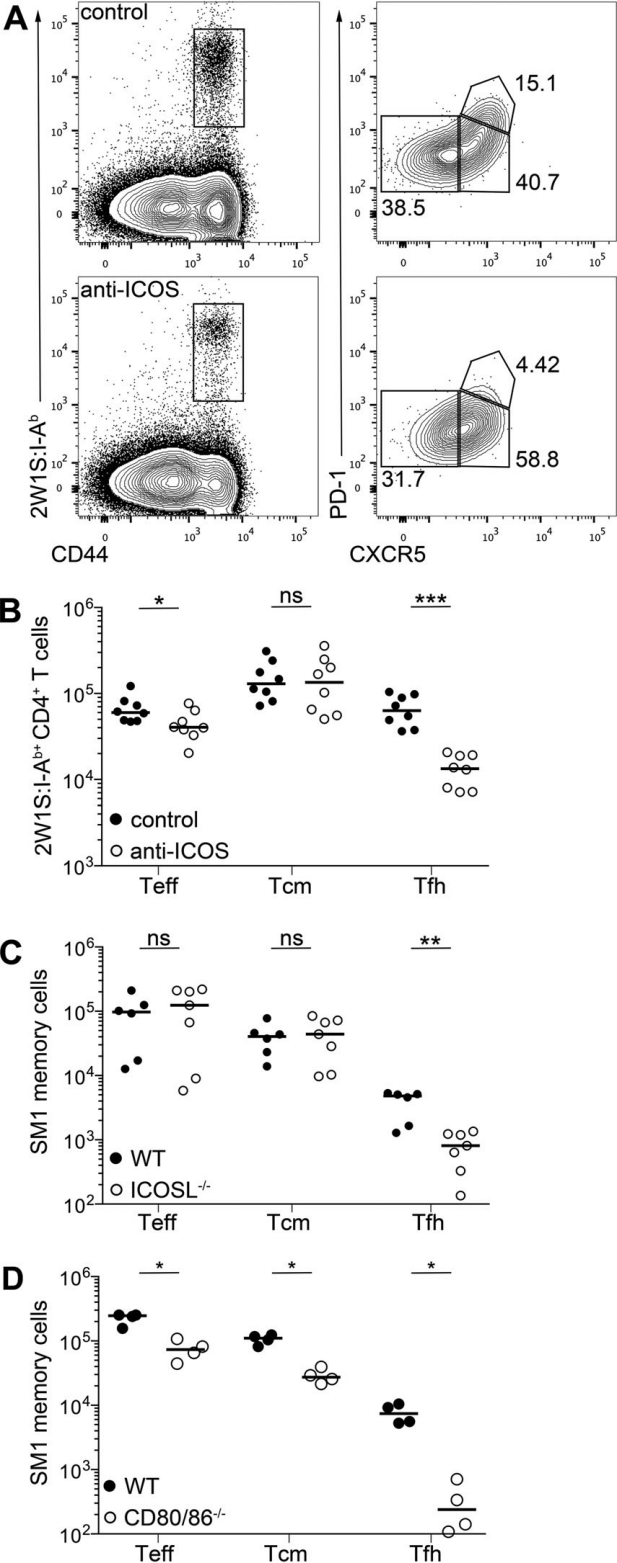
ICOS is required for expansion of antigen‐specific Tfh cells post secondary challenge. (A and B) WT mice were infected with Lm‐2W1S then challenged again at 28 dpi and treated with anti‐ICOS or isotype control mAb 1 day pre‐ and post secondary challenge with Lm‐2W1S. (A) Three days post secondary challenge CD44^hi^ 2W1S:I‐A^b+^ CD4^+^ T cells were stained for CXCR5 and PD‐1 expression and analyzed by flow cytometry. (B) CD44^hi^ 2W1S:I‐A^b+^ CD4^+^ CXCR5^−^ (Teff), CXCR5^+^ (Tcm precursors), and CXCR5^+^PD‐1^+^ (Tfh) cells were enumerated 3 days post secondary challenge. (C and D) Mice were injected with SM1 memory cells and challenged 28 days posttransfer. Enumeration of SM1 memory cells that are CXCR5^−^ (Teff), CXCR5^+^ (Tcm precursors), and CXCR5^+^PD‐1^+^ (Tfh) cells from (C) ICOSL^−/−^ and (D) CD80^−/−^CD86^−/−^ mice day 4 post secondary challenge. (A) Plots are representative of ≥6 mice from two independent experiments. Values on plots are percentages. (B, C) Graphs show pooled data or (D) representative data from two independent experiments. Each data point represents one mouse; bars show medians. Mann–Whitney test: ns *p* > 0.05, *p < 0.05, **p ≤ 0.01, ***p ≤ 0.001.

Our data indicated that signals through ICOS were critical for the generation of both Tcm and Tem populations, rather than the subsequent survival of these cells during the memory phase of the response. To assess the requirement for ICOS signals upon secondary challenge mice were infected with Lm‐2W1S and then again at 28 dpi and analyzed 3 days later. Blocking anti‐ICOS or isotype control mAb were administered day 1 pre‐ and postsecondary challenge. Mice receiving blocking anti‐ICOS mAb showed substantially impaired Tfh cell formation, modestly impaired Tem populations and no significant effect on Tcm cells (Fig. 4A and B). To further assess the contribution of costimulatory molecules only at secondary challenge, SM1 memory cells generated in WT mice were transferred into ICOSL^−/−^ or WT hosts that were then challenged 28 days later with Lm‐Flic. While expansion of Tcm and Tem cells was unaffected, again the number of number of Tfh cells was substantially reduced indicating that this cell type remains heavily ICOS dependent (Fig. 4C). In contrast, transfer of SM1 memory cells into CD80^−/−^CD86^−/−^ hosts subsequently challenged with Lm‐Flic revealed a significant reduction in all of the T‐cell subsets analyzed, demonstrating that these populations remain partially dependent on CD28 signals for expansion (Fig. 4D).

### Combination of CTLA4Ig and anti‐ICOS mAb enhances blockade of antigen‐specific CD4^+^ T‐cell response

**Figure 5:**
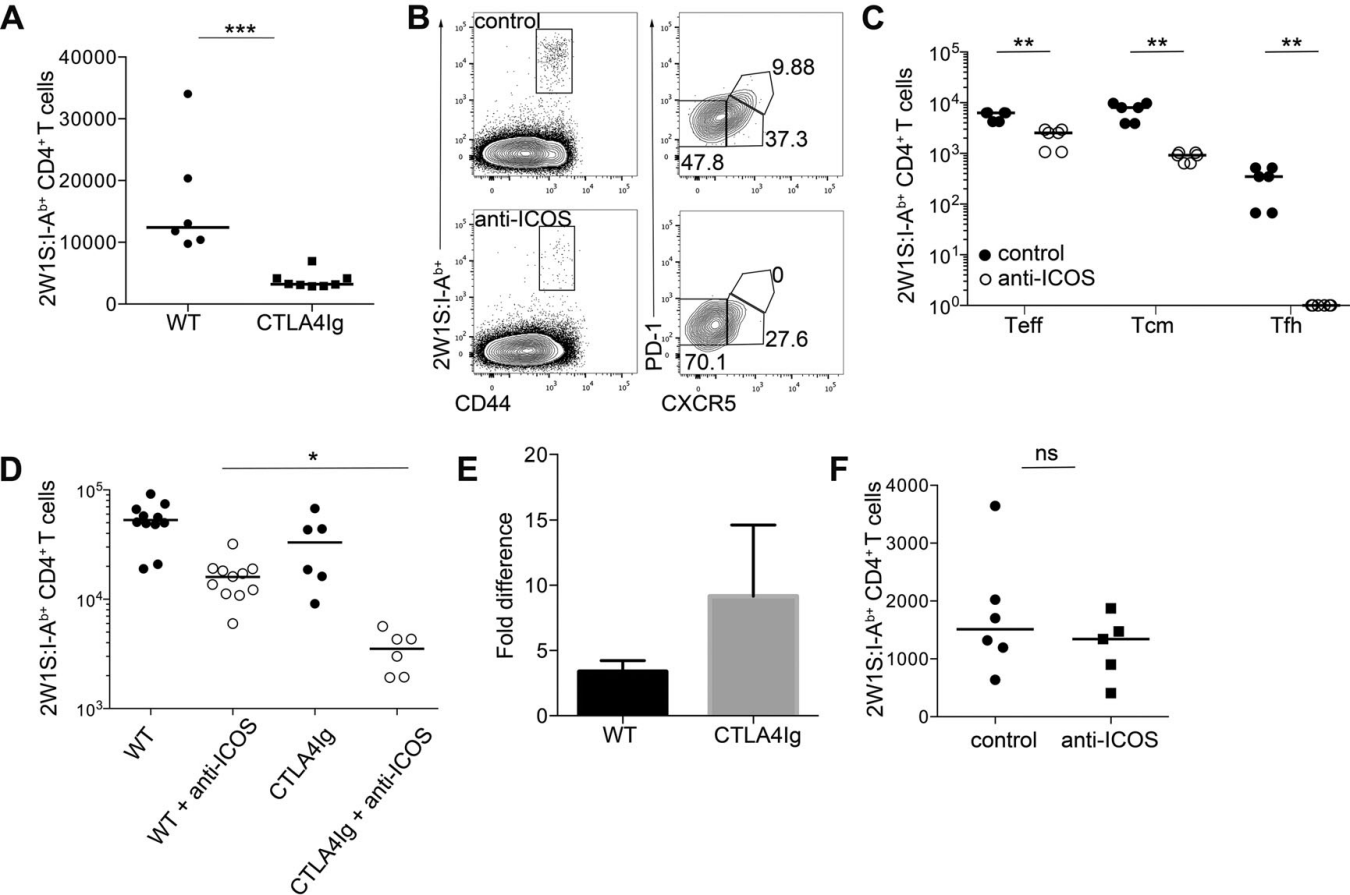
Therapeutic combination of blocking anti‐ICOS mAb and CTLA4Ig enhances blockade of CD4^+^ T‐cell response. (A) WT and CTLA4Ig mice were infected with Lm‐2W1S. CD44^hi^ 2W1S:I‐A^b+^ CD4^+^ memory T cells were enumerated 28 dpi by flow cytometry. (B and C) CTLA4Ig mice were infected with Lm‐2W1S and treated with anti‐ICOS or isotype control mAb 0 and 3 dpi. (B) CD44^hi^ 2W1S:I‐A^b+^ CD4^+^ T cells were stained for CXCR5 and PD‐1 expression 7 dpi and analyzed by flow cytometry. Plots are representative of ≥6 mice from three independent experiments. Values on plots are percentages. (C) CD44^hi^2W1S:I‐A^b+^CD4^+^ CXCR5^−^ (Teff), CXCR5^+^ (Tcm precursors), and CXCR5^+^PD^−^1^+^ (Tfh) cells were enumerated 7 dpi from control and anti‐ICOS treated mice. (D) CD44^hi^ 2W1S:I‐A^b+^ CD4^+^ T cells were enumerated 7 dpi from WT and CTLA4Ig mice treated with anti‐ICOS or isotype control mAb 0 and 3 dpi. Kruskal–Wallis one‐way ANOVA with post hoc Dunn's test: *p ≤ 0.05. (E) Fold difference between mice treated with anti‐ICOS or isotype control mAb in number of CD44^hi^ 2W1S:I‐A^b+^ CD4^+^ T cells in WT and CTLA4Ig mice. Bars show mean + SEM of fold difference between experiments. (F) CD44^hi^ 2W1S:I‐A^b+^ CD4^+^ memory T cells were enumerated from CTLA4Ig mice 28 dpi with Lm‐2W1S, treated with blocking anti‐ICOS or isotype control mAb twice weekly for 4 weeks. (A, C–F) Graphs show pooled data from ≥2 independent experiments. Values of 0 were assigned as 1. Each data point represents one mouse; bars show medians. Mann–Whitney test: ns *p* > 0.05, **p ≤ 0.01, ***p ≤ 0.001.

Antibody‐mediated blockade of ICOS signals during the primary response comprehensively blocked Tfh cell formation and significantly impacted on the resulting memory population, although the total number of antigen‐specific CD4^+^ T cells at day 7 was only modestly reduced. Blocking anti‐ICOS mAb caused a greater reduction in the memory cell population at day 28 (approximate tenfold reduction versus WT controls, Fig. 2B) than observed in CTLA4Ig mice, where the fold difference was approximately 4 (Fig. 5A). Given the modest reduction in Teff and Tcm precursors observed in the CTLA4Ig mice at day 7, we sought to assess the effects of combined blockade of ICOS and CD28 signals as a dual therapeutic approach. Therefore CTLA4Ig mice were treated with blocking anti‐ICOS or isotype control mAb at 0 and 3 dpi with Lm‐2W1S. At 7 dpi there was a reduction in all 2W1S‐specific T‐cell subsets in CTLA4Ig mice that received blocking anti‐ICOS treatment compared to control treated CTLA4Ig mice and Tfh cells were absent (Fig. 5B and C). Interestingly, there was an approximate 8.5‐fold reduction in 2W1S‐specific CD4^+^ T cellsin CTLA4Ig mice treated with anti‐ICOS mAb compared to a 3.5‐fold reduction in WT mice indicating that combined CTLA4Ig and anti‐ICOS treatment enhances blockade of the T‐cell response (Fig. 5D and E).

Finally, given that blockade of ICOS or CD28 signals alone did not affect memory cell survival, we sought to test whether this reflected redundancy in the requirement for these signals at this time. Therefore, CTLA4Ig mice were infected with Lm‐2W1S and blocking anti ICOS or isotype control mAb were administered for 4 weeks starting 28 dpi. Enumeration of 2W1S‐specific CD4^+^ memory T cells revealed no significant difference, indicating that rather than redundancy in the memory cell requirements for CD28 and ICOS signals, neither are necessary for survival at this stage of the response (Fig. 5F).

### Discussion

Studies on the importance of ICOS signals for CD4^+^ T‐cell responses have focused on the clear requirement for this molecule in the formation of Tfh cells and T‐dependent Ab responses 24, 25, 31. Here, we have dissected the different stages of a CD4^+^ T‐cell response and assessed the exact time at which ICOS signals are required for the different subsets of an endogenous CD4^+^ T‐cell population. While CD28 signals were critical for the early expansion of CD4^+^ T cells, the subsequent formation of a robust memory cell pool was highly dependent on signals through both CD28 and ICOS and mAb blocking ICOS during priming effectively reduced memory cell numbers. Furthermore, we show that in this systemic response, both the Tcm and Tem populations require ICOS for their generation, but not their subsequent survival nor initial expansion upon secondary challenge.

Members of the TNF receptor super family such as OX40 have been intensely studied as key signals in the survival of CD4^+^ T cells 35 and signals through these molecules are important in the survival and function of Teff cells 36, 37. While requirements for costimulatory molecules likely depend upon the nature of the infection and the type or quantity of antigen, a striking feature of this study was the very brief expression of OX40 only at times of antigen exposure 14. In contrast, responding 2W1S‐specific CD4^+^ T cells retain expression of ICOS throughout the phases of the response, an observation that prompted us to dissect when signals though this molecule might be important. Several previous studies have indicated a requirement for ICOS only for CD4^+^ Tem cells and these have been reported to express the highest levels of ICOS compared to Tcm and naive T cells 29. Although these studies have relied upon a surface phenotype, which does not distinguish responding cells from memory phenotype cells known to have different survival requirements 12, studies with antigen‐specific CD4^+^ T cells also indicated that only Tem cells depend on ICOS for survival 30. The data presented here clearly show that in the response to Lm‐2W1S infection, generation of all memory cells was heavily dependent upon ICOS signals, which is consistent with observations from human patients with ICOS deficiency 28. Interestingly, studies tracking HA‐specific CD4^+^ memory cells using MHCII tetramers following i.n. infection with influenza revealed no significant differences in the total memory cell population in ICOS^−/−^ mice, with a reduced Tem population balanced by an expanded number of Tcm cells 30. Furthermore, Mahajan et al. concluded that following subcutaneous immunization with H19‐Env peptide emulsified with CFA, ICOS signals were not required for memory cell survival, but were important upon restimulation 26. When compared with the data presented here, these studies indicate that the importance of ICOS signals in a CD4^+^ T‐cell response may be dictated by the route of antigen encounter, for example i.v. or i.n. infection with Lm‐2W1S results in very different memory cell responses 1. Furthermore, responses generated within mucosal secondary lymphoid tissue may involve distinct costimulatory requirements, which should be considered when immunizing at such sites or attempting to block autoimmune T cells in such tissues. Different adjuvants or types of infection will also likely shape the costimulatory profile of the response, through the stimulation of distinct PRR. Our experiments have focused on the response to Lm‐2W1S and previous studies have identified an important role for ICOS signals in the response to *L. monocytogenes* and other intracellular bacteria 38, 39. Finally, it is not clear whether the costimulatory requirements of memory cells upon secondary challenge depend upon the nature of the initial response, which may explain the differences between data presented here and in other studies 26.

Temporary blockade of ICOS during the primary response had substantial effects on the resulting memory population, not dissimilar to that observed in ICOSL^−/−^ mice and more impressive than in CTLA4Ig mice, demonstrating the therapeutic potential of targeting this pathway to limit CD4^+^ T‐cell responses. Since both genetic deficiency in ICOSL or Ab‐mediated blockade of ICOS only modestly reduced the total number of antigen‐specific CD4^+^ T cells at the peak of the primary response, this would indicate that either signals through ICOS are required beyond 7 dpi or the absence of these signals during the primary response caused the survival of the CD4^+^ T cells to be grossly impaired. Although the blocking anti‐ICOS mAb has a half‐life of approximately 7–10 days in vivo (G. Carlesso, unpublished observation), systematic blockade of ICOS signals at different time points in the response is really required to dissect this further. While CD28 signals are clearly crucial for the initiation of CD4^+^ T‐cell responses and lie upstream of ICOS expression 17, therapeutic targeting of CD28 signals using abatacept has yielded mixed results clinically in blocking CD4^+^ T‐cell responses. Abatacept treatment has shown promise in autoimmune conditions such as rheumatoid arthritis and type 1 diabetes, but not multiple sclerosis or ulcerative colitis 40. In transplantation a modified version of abatacept has been used, named belatacept, which more effectively inhibited renal transplant rejection 40, 41, 42. Blockade of ICOS signals has also been trialed clinically and been shown to prolong cardiac allograft survival 43, 44. To further enhance costimulatory blockade combined targeting of multiple pathways has been proposed 40, 45 and inhibition of both the ICOS and CD40 ligand pathways resulted in allograft tolerance and prevented autoimmune diabetes in NOD mice 46. Here, we show that whilst CTLA4Ig mice produce 10–30 μg/mL serum levels of CTLA4Ig protein 33 and GC development is blocked, the effect on other T‐cell subsets was surprisingly modest compared to CD80^−/−^CD86^−/−^ mice. Our data show that while signals through CD28 are clearly critical in the generation of CD4^+^ T‐cell responses, CTLA4Ig at the levels maintained in these mice does not seem to provide a robust blockade of CD80 and CD86. Strikingly, provision of only two doses of anti‐ICOS mAb was sufficient to substantially reduce the resulting memory T‐cell population, while modestly affecting the primary response, recapitulating data from ICOSL^−/−^ mice. The combined use of CTLA4Ig and anti‐ICOS mAb in our model was far superior in blocking primary CD4^+^ T‐cell responses, suggesting this dual targeting may have significantly enhanced clinical benefit in treating autoimmunity and graft rejection.

Whilst the cytokines IL‐7 and IL‐15 have been demonstrated to affect memory CD4^+^ T‐cell survival, a role for costimulatory molecules remains unclear. To test a possible contribution from CD28 or ICOS signals we generated antigen‐specific memory CD4^+^ T cells in vivo within WT hosts and subsequently transferred these into ICOSL^−/−^ or CD80^−/−^CD86^−/−^ mice. The lack of these ligands had no significant effect on the number of memory CD4^+^ T cells subsequently recovered indicating that neither CD28 nor ICOS signals are required solely for memory cell survival within the timescale of these studies. Interestingly, this memory cell transfer model indicated that CD4^+^ memory T cells remain dependent on CD28 signals for optimal expansion upon secondary challenge, consistent with studies of memory cell recall using CTLA4Ig fusion proteins 47. ICOS signals remain important for the generation of Tfh cells upon challenge but had no clear effect on the expansion of Tem and Tcm cells at this time point. The modest reduction in Tem cells after challenge observed with 2W1S‐specific CD4^+^ T cells in ICOSL^−/−^ mice was not observed with SM1 cells, potentially reflecting differences between endogenous and TCR transgenic T cell responses.

In summary, beyond the formation of Tfh cells, signals through ICOS appear critical for the generation of a functional pool of Tem and Tcm cells following acute bacterial infection. The effectiveness of anti‐ICOS blocking mAb in this response suggests that therapeutic targeting of this pathway may substantially improve clinical attempts to control CD4^+^ T cells, perhaps in combination with blockade of CD28 signals.

## Materials and methods

### Mice

Mice used were BoyJ, C57BL/6 (obtained from Harlan or in house), ICOSL^−/−^, CD80/86^−/−^, CTLA4Ig 33, Rag^−/−^ SM1 (CD45.1^+^CD45.2^+^). Mice were only compared to those that had been bred in the same facility. Animals were bred in accordance with Home Office guidelines at the University of Birmingham, Biomedical Services Unit.

### Cell transfer

A total of 10^4^ SM1 cells harvested from spleen and peripheral lymph nodes of TCR transgenic Rag^−/−^ SM1 mice were injected i.v. into C57BL/6 or BoyJ mice before i.v. infection with Lm‐Flic the following day 48. For memory cell transfers, secondary lymphoid tissues were harvested 28 dpi and cells were enriched using MACS columns and transferred i.v. into hosts 32. Cells were transferred into twice as many recipients as donors, typically approximately 2.5 × 10^4^ memory SM1 cell were transferred.

### Infection

Mice were infected i.v. with 10^7^ ActA‐deficient Lm‐2W1S (kind gift from Dr. Marc Jenkins) or Lm‐Flic (kind gift from Dr. Sing Sing Way) as described 1, 14. Mice were challenged at 28 dpi or post SM1 memory cell transfer and mice were sacrificed on day 3 or 4 post secondary challenge, respectively. For in vivo restimulation of memory cells mice were injected i.v. with 100 μg 2W1S peptide and 2.5 μg LPS 28 dpi and analyzed after 4 h.

### Antibody injection

Blocking anti‐ICOS and isotype control mAb were provided by MedImmune 49, 50. Mice were injected i.p. with 0.25 mg anti‐ICOS or isotype control mAb. Mice were injected 0 and 3 dpi for primary responses, 0 and 4 dpi for memory responses and 1 day pre‐ and post secondary challenge. To assess memory cell survival mice were injected twice weekly for 4 weeks from 28 dpi.

### Flow cytometry

CD44^hi^ 2W1S:I‐A^b+^ CD4^+^ T cells were enumerated from spleen at 7 dpi and a pool of spleen and LN for all memory responses. Staining with PE conjugated 2W1S:I‐A^b^ was carried out as described previously 14. For in vivo restimulation experiments 10 μg brefeldin A was added. Cell surface staining was done at 4°C for 30 min, except for CXCR5 that was stained for 1 h at room temperature. Enrichment for 2W1S‐specific memory T cells was performed, as described previously, using anti PE MicroBeads (Miltenyi Biotech) and MACS enrichment 51. Samples were run using a Fortessa X20 (BD Biosciences) and analyzed using FlowJo software (Tree Star). For intracellular cytoplasmic staining, cells were fixed and permeabilized with Cytofix/Cytoperm Plus (BD). Intracellular cytokines were stained with IL‐2 488 and IFN‐γ PECy7 (BD Biosciences). Ten microliters of Spherotech Accucount blank particles was used to calculate cell frequency.

### Statistics

Data were analyzed using GraphPad Prism (version 6.0e). Nonparametric Mann–Whitney test was used to determine significance which was set at *p* ≤ 0.05. Kruskal–Wallis one‐way ANOVA was used to compare multiple groups with post hoc Dunn's test where stated. Median values were calculated and used in all analyses except where stated.

## Conflict of interest

R. Herbst and G. Carlesso are full‐time employees of MedImmune LLC.

**Abbreviations**
dpi: days post infection
ICOSL: ICOS ligand
Lm‐2W1S: *Listeria monocytogenes* expressing 2W1S peptide
Tcm: T central memory
Tem: T effector memory
Tfh: T follicular helper

## Supporting information

As a service to our authors and readers, this journal provides supporting information supplied by the authors. Such materials are peer reviewed and may be re‐organized for online delivery, but are not copy‐edited or typeset. Technical support issues arising from supporting information (other than missing files) should be addressed to the authors.

Figure s1
Figure s2
