# Supplementary material for: ICOS is required for the generation of both central and effector CD4+ memory T‐cell populations following acute bacterial infection
Source: Eur J Immunol. 2015 Apr 7;45(6):1706–15. doi: 10.1002/eji.201445421 (PMC4736665; doi:10.1002/eji.201445421)
Supplement: Supplementary file 1 — Figure s1 Figure s2 [file EJI-45-1706-s001.pdf]

Supporting Information Figure 1

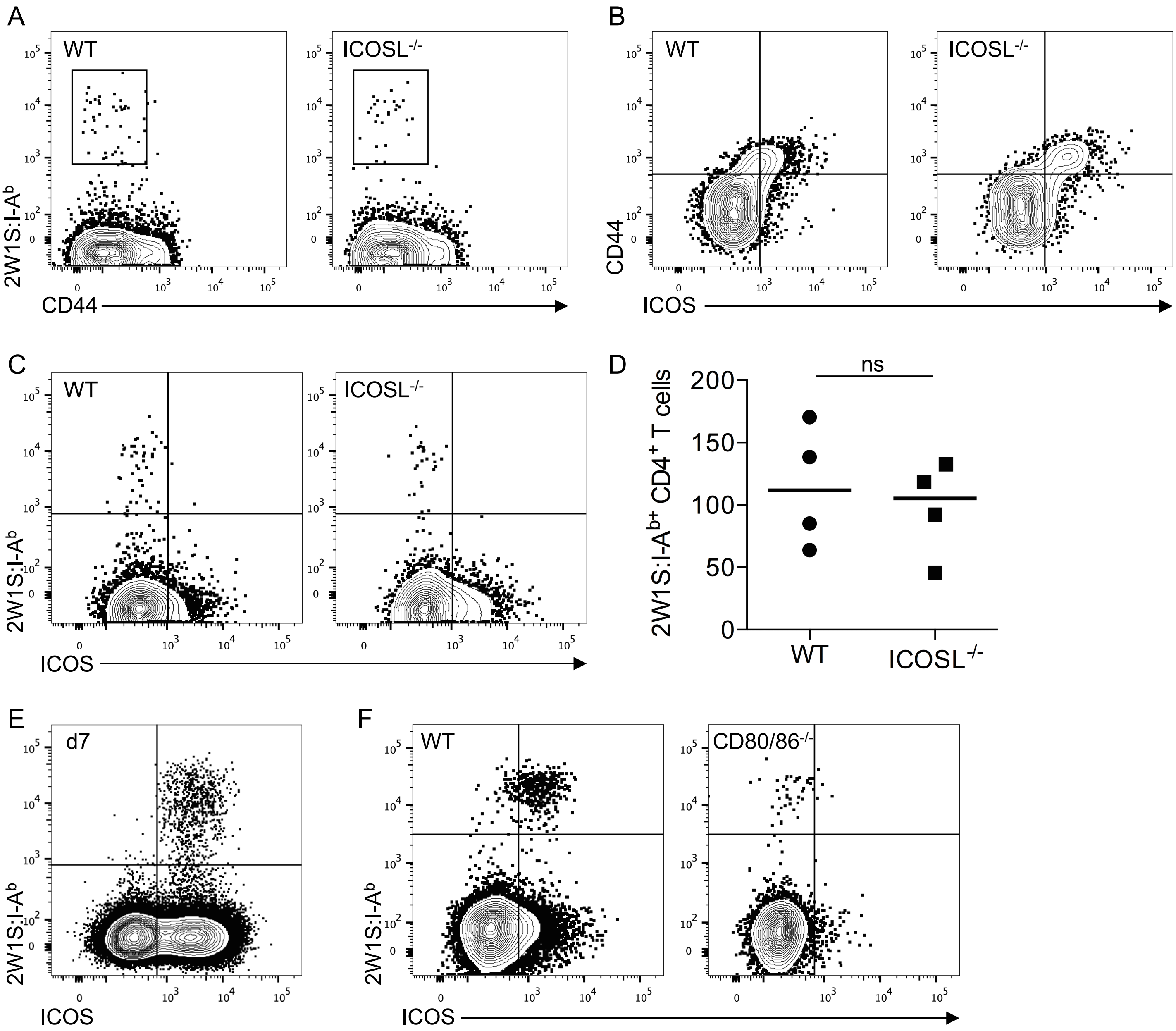

Supporting Information Figure 2

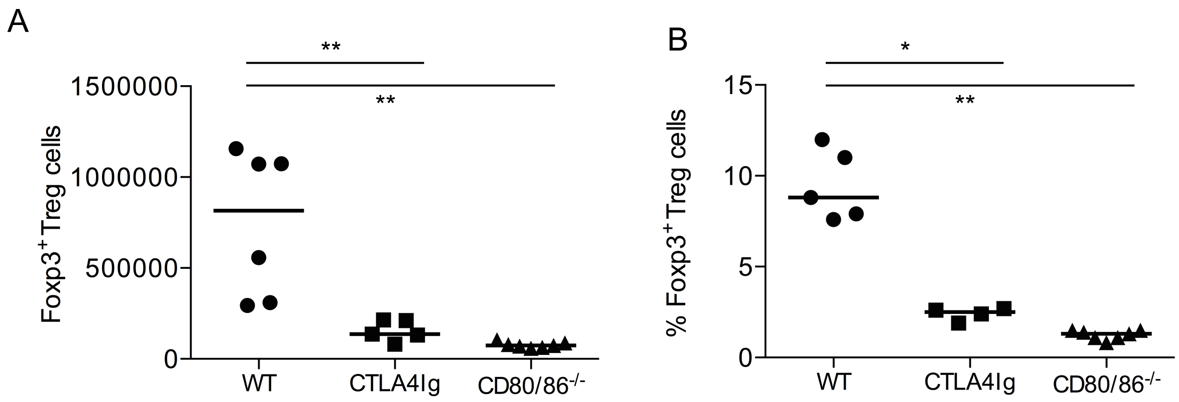

Supporting Information Figure Legends

Supporting Information Figure 1

(A) Expression of CD44 on 2W1S:I-A^{b+} $CD4^+$ T cells from naive WT and $ICOSL^{-/-}$ mice. Plots are gated on $CD4^+$ T cells; show 2 mice pooled and are representative of 4 mice. (B) Expression of CD44 and ICOS by $CD4^+$ T cells from naive WT and $ICOSL^{-/-}$ mice. Plots are representative of 4 mice. (C) Expression of ICOS on 2W1S:I-A^{b+} $CD4^+$ T cells from naive WT and $ICOSL^{-/-}$ mice. Plots are gated on $CD4^+$ T cells; show 2 mice pooled and are representative of 4 mice. (D) 2W1S:I-A^{b+} $CD4^+$ T cells from naive WT and $ICOSL^{-/-}$ mice were enumerated by flow cytometry. Graph shows pooled data from 2 independent experiments. Each data point represents one mouse; bars show medians. Mann-Whitney test: ns $p>0.05$. (E) Expression of ICOS on 2W1S:I-A^{b+} $CD4^+$ T cells 7 dpi with Lm-2W1S in WT mice. Plots are gated on $CD4^+$ T cells and are representative of ≥6 mice. (F) Expression of ICOS on 2W1S:I-A^{b+} $CD4^+$ T cells WT and $CD80^{-/-}CD86^{-/-}$ 28 dpi with Lm-2W1S. Plots are gated on $CD4^+$ T cells and are representative of ≥6 mice.

Supporting Information Figure 2

(A) Treg cells from WT, CTLA4IG and $CD80^{-/-}CD86^{-/-}$ mice were enumerated by flow cytometry. (B) Percentage of Treg cells among $CD4^+$ T cells from WT, CTLA4IG and $CD80^{-/-}CD86^{-/-}$ mice. Graphs show pooled data from ≥2 independent experiments. Each data point represents one mouse; bars show medians. Mann-Whitney test: *$p\leq0.05$, **$p\leq0.01$.
